# Volatile organic compounds produced by *Bacillus velezensis* L1 as a potential biocontrol agent against postharvest diseases of wolfberry

**DOI:** 10.3389/fmicb.2022.987844

**Published:** 2022-08-24

**Authors:** Lijun Ling, Hong Luo, Caiyun Yang, Yuanyuan Wang, Wenting Cheng, Mingmei Pang, Kunling Jiang

**Affiliations:** ^1^College of Life Science, Northwest Normal University, Lanzhou, China; ^2^Bioactive Products Engineering Research Center for Gansu Distinctive Plants, Northwest Normal University, Lanzhou, China; ^3^New Rural Development Research Institute, Northwest Normal University, Lanzhou, China

**Keywords:** volatile organic compounds, *Bacillus velezensis* L1, *Alternaria iridiaustralis*, wolfberry disease, biological control

## Abstract

Volatile organic compounds (VOCs) produced by antagonistic microorganisms have good biocontrol prospects against postharvest diseases. Infection caused by *Alternaria iridiaustralis* and 10 other significant fungal diseases can be successfully inhibited by VOCs produced by an identified and screened endophytic strain L1 (*Bacillus velezensis*). This study revealed the *in vivo* and *in vitro* biocontrol effects of VOCs released by *B. velezensis* L1 on *A. iridiaustralis*, a pathogenic fungus responsible for rot of wolfberry fruit. The inhibition rates of VOCs of *B. velezensis* L1 on the mycelial growth of *A. iridiaustrali*s *in vitro* were 92.86 and 90.30%, respectively, when the initial inoculum concentration on the plate was 1 × 10^9^ colony forming unit (CFU)/ml. Spore germination and sporulation were 66.89 and 87.96%, respectively. VOCs considerably decreased the wolfberry’s disease index and decay incidence *in vivo*. Scanning electron microscopy revealed that the morphological and structural characteristics of *A. iridiaustralis* could be altered by VOCs. Ten VOCs were identified through headspace-gas chromatography-ion mobility spectrometry. Pure chemical tests revealed that 2.3-butanedione had the strongest antifungal effects, totally inhibiting *A. iridiaustralis* in wolfberry fruit at a 60 μl/L concentration. The theory underpinning the potential application of VOCs from *B. velezensis* is provided herein. This is also the first study to document the antifungal capabilities of the *B. velezensis* strain on postharvest wolfberry fruit.

**GRAPHICAL ABSTRACT:**
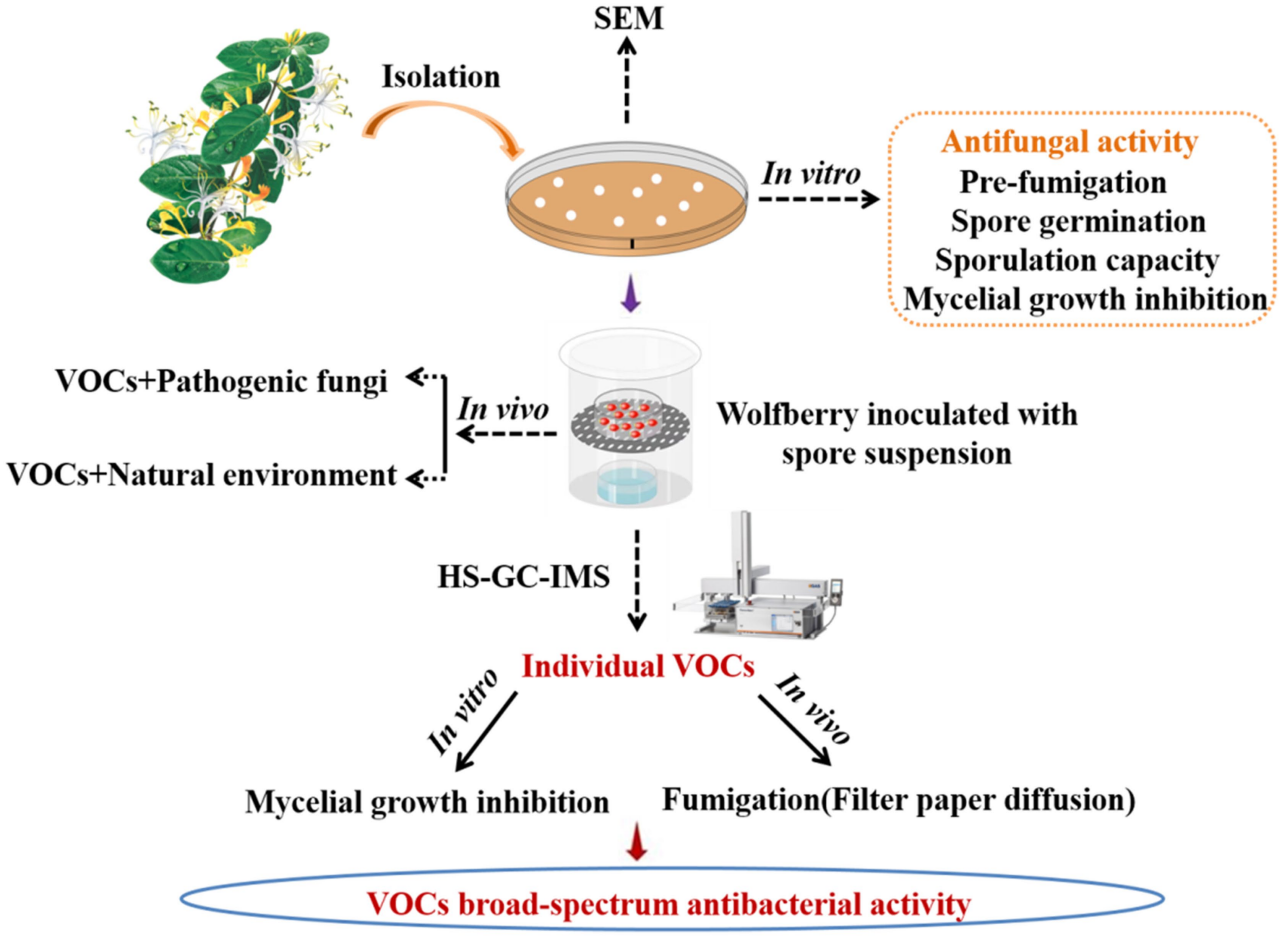

## Introduction

Wolfberry (*Lycium barbarum* L) is gradually becoming one of the most popular functional fruits worldwide as it is rich in nutrients and phytochemicals (Wenli et al., 2021). Wolfberry has anticancer, antioxidant, antiaging, blood sugar control, and immune-enhancing properties. Therefore, this fruit is widely used in food and medicine fields (Zhou et al., 2017; Yajun et al., 2019). However, wolfberry fruit is highly perishable because of its susceptibility to various postharvest pathogenic diseases. Fungal diseases are the main cause of postharvest losses of wolfberry fruits. Particularly, *Alternaria* sp. are the most common postharvest pathogenic fungi of wolfberry fruits and can significantly reduce the fruit value and shelf life, causing severe economic losses (Yuan et al., 2012; Wei et al., 2017).

Regarding postharvest diseases of fruits, traditional physical methods can delay senescence but cannot completely prevent rot, which ultimately reduces fruit quality (Alijani et al., 2019). On the other hand, chemical fungicides have a substantial inhibitory effect on pathogenic fungi, but their long-term and large-scale use leads to deposition of residues in the environment and resistance of pathogenic fungi (Mari et al., 2016). Therefore, finding new, safe, efficient, and environmentally friendly methods for controlling these diseases is of great significance.

Increasing number of researchers have recently focused on biological control agents (BCAs; Do Kim et al., 2020). According to research, many bacteria can produce volatile organic compounds (VOCs) with antifungal function, which have shown strong inhibitory effects on fungal diseases (Di Francesco et al., 2020a). Gotor-Vila et al. (2017) reported that VOCs produced by *Bacillus amyloliquefaciens* CPA-8 significantly inhibited the mycelial growth of postharvest pathogens and reduced the rot of cherry fruits. Arrarte et al. (2017) revealed that VOCs released by *Candida sake* can control decay of apples caused by postharvest pathogens. Similarly, Oro et al. (2018) found that VOCs from *Wickerhamomyces anomalus*, *Metschnikowia pulcherrima*, and *Saccharomyces cerevisiae* can inhibit pathogenic fungi that cause rot in strawberries and control postharvest diseases. In this way, VOCs released by BCAs provide a new solution for the prevention and treatment of postharvest diseases.

VOCs have the characteristics of strong permeability and high diffusion efficiency, and are easy degradation, can greatly reduce residues. In a relatively closed environment, a full range of biological fumigation can be performed on items to achieve better results (Qin et al., 2017; Toffano et al., 2017). Therefore, compared with chemical fungicides, the mode of action of VOCs produced by BCAs is safer and more efficient. *B. velezensis* has been widely studied because of its highly effective inhibitory effect on pathogenic microorganisms. Li et al. (2020) reported that VOCs from *B. velezensis* CT32 could inhibit strawberry vascular wilt pathogens *Verticillium dahliae* and *Fusarium oxysporum*. Gao et al. (2017) indicated that VOCs emitted by *B. velezensis* ZSY-1 could inhibit *A. solani* and *Botrytis cinerea*. Calvo et al. (2020) showed that VOCs produced by *B. velezensis* strains BUZ-14, I3 and I5 could inhibit postharvest fungal pathogens of fruits, thereby reducing the disease severity in grapes and apricots. *B. velezensis* is considered a promising BCA against postharvest fungal diseases.

Currently, research on postharvest preservation of wolfberry fresh fruit fruits is lacking. Therefore, this study evaluated the *in vivo* and *in vitro* effects of VOCs produced by *B. velezensis* L1 and individual pure substance against postharvest pathogenic fungi *A. iridiaustralis* in wolfberry fruit.

## Materials and methods

### Fruit and plants

The wolfberry fruit was purchased from Ningxia, Gansu. Fully ripe fruits of the same size and without any mechanical damage were sterilized with 1% sodium hypochlorite, rinsed three times with sterile distilled water (SDW), and air-dried naturally on an ultra-clean bench.

*Lonicera maackii* (Rupr.) Maxim is from Lanzhou Botanical Garden, Gansu, China. Picked stems and leaves were stored in airtight bags and kept at 4°C for later use.

### Culture media and microorganisms

Tianqi Gene Biotechnology Co., Ltd. provided Luria–Bertani medium (LB) and potato dextrose agar medium (PDA; Lanzhuo, China). Agar was purchased from Niuniu Biochemical Co., Ltd. (Lanzhuo, China).

Wolfberry fungal pathogen *A. iridiaustralis* LB7 (MN 944921) was supplied by the College of Life Sciences, Northwest Normal University, China’s Plant-Microbe Interactions Research Lab. This pathogen has been demonstrated to significantly worsen postharvest wolfberry deterioration in our previous study (Ling et al., 2021). *A. iridiaustralis* was inoculated on PDA at 28°C for 7 days, the fungal spores were resuspended in SDW, and the concentration of the spore suspension was adjusted to 1 × 10^5^ spores/mL with a hemocytometer.

Pathogenic fungi *Phytophthora capsici* (ACCC 37132), *Colletotrichum capsici* (ACCC 36946), *F. oxysporum* (ACCC 39326), *B. cinerea* (ACCC 37347), *Rhizoctonia solani* (ACCC 38870), *F. graminearum* (ACCC 39334) were obtained from Agricultural Culture Collection of China, Beijing. *F. annulatum* (MT 434004), *Talaromyces tumuli* (MT 434003), *Colletotrichum fioriniae* (MN 944922), and *F. arcuatisporum* (MN 944920) were isolated from disease plants and all pathogenic strains were stored in the laboratory (Ling et al., 2021).

### Isolation, screening and identification of a highly antifungal bacterial endophyte

Using our previous technique, we isolated and characterized endophytic bacteria from *L. maackii* (Rupr.) Maxim (Ling et al., 2019). Briefly, the fresh stems and leaves stored in the refrigerator were removed, rinsed under running water, air-dried naturally, sterilized, and rinsed three times with sterile water. Next, the stems and leaves were cut using a sterile scalpel and cultured in solid LB medium plates at 37°C. Each colony was transferred to new medium until a pure culture of the strain is obtained. Using the two sealed base plate approach, the strain with the greatest capacity to suppress the pathogenic fungus was selected for further investigation. In general, 80 μl of bacterial suspension was evenly distributed on LB solid medium. Subsequently, a 6 mm-diameter fungal disk was cut and placed in the center of a sterile PDA petri dish. The two plates were sealed to prevent the loss of VOCs. The bacteriostatic ability of endophytic bacteria against pathogenic fungi was evaluated on the basis of the diameter of the fungus.

The bacterial DNA template obtained by screening was extracted using of the bacterial genome extraction kit (Huada Gene Co., Ltd., Wuhan, China). The 16S rDNA fragment was amplified through PCR by using universal primers 27F (5′AGAGTTTGATCCTGGCTCAG3′) and 1429R (5′GGTTACCTTGTTACGACTT3′). The cycle parameters were as follows: predenaturation at 96°C for 5 min, followed by 30 cycles of denaturation at 96°C for 30 s, annealing at 62°C for 30 s, and a final extension at 72°C for 30 s. The extension was performed at 72°C for 10 min at the end of the cycle. PCR products were detected through 1% agarose gel electrophoresis and sequenced. The sequencing results of 16S rDNA were input into the nucleic acid database alignment system of the National Center for Biological Information (NCBI) website, and nucleic acid sequence alignment analysis was performed using the Blast program. A Phylogenetic tree was constructed using the neighbor-joining method with MEGA-X software to analyze the phylogenetic relationship.

### *In vitro* inhibitory activity of VOCs from *Bacillus Velezensis* L1

#### Inhibitory activity of VOCs with different inoculation concentrations of *Bacillus Velezensis* L1

A dual culture method was used to evaluate the inhibitory effect of antagonistic bacteria produced VOCs on *A. iridiaustralis* mycelial growth and conidial germination (Ye et al., 2020; Di Francesco et al., 2020b). SDW was used to inoculate the control plates with the same amounts of bacteria as the test plates, which was 80 μl of bacterial suspension at a concentration of 10^6^–10^9^ colony forming units (CFU)/ml. The plate cover was then changed out by a PDA plate that had been injected with a 6 mm-diameter disk or 20 μl of *A. iridiaustralis* spore suspension. Both plates were immediately sealed with parafilm, and cultured for 7 days at 28°C. Each experiment was conducted three times.

#### Effect of VOCs from *Bacillus velezensis* L1 on the spore germination and sporulation capacity of *Alternaria iridiaustralis*

With a few minor adjustments, the preceding approach was used to measure spore germination and sporulation capacity (Bu et al., 2021; Wang et al., 2021b). LB plates were inoculated with strain L1 and PDA plates were covered with a fungal spore suspension (1 × 10^5^ spores/mL), as indicated earlier. After 12 h incubation at 28°C, spore germination could be visualized under a light microscope. The sporulation capacity experiment was conducted according to instructions provided in Section 2.4.1. In general, a 6 mm fungal plug was removed from the cultivated PDA medium and vortexed in SDW. A hemocytometer was used to count the spores.

#### Effect of VOC prefumigation on mycelium growth

The LB plate coated with the 80 μl bacterial suspension and the PDA plate containing a 6 mm-diameter disk were snap-sealed. After 4 or 5 days of fumigation in the incubator, 6 mm-diameter disks were cut from the PDA plate by using a punching bear, transferred to a new PDA plate, and incubated at 28°C for 7 days. Then, mycelial growth of pathogenic fungi were measured.

#### Scanning electron microscopy analysis

The effect of VOCs produced by *B. velezensis* L1 on pathogenic fungi was observed using scanning electron microscopy (SEM). SEM analysis was performed as described previously (Wonglom et al., 2020).

### *In vivo* biocontrol of *Alternaria iridiaustralis* on wolfberry fruit by VOCs from *Bacillus velezensis* L1

#### Inhibitory effects of VOCs on *Alternaria iridiaustralis in vivo*

The biocontrol effects of different initial amounts of VOCs *in vivo* were investigated using different numbers of plates. LB plates were treated with 80 μl bacterial suspension (1 × 10^8^ CFU/ml), and these uncovered plates were then placed at the bottom of sterile glass containers in numbers of 2, 4, 6, and 8, respectively. Then, 5 μl of *A. iridiaustralis* spore suspension was inoculated into wolfberry fruits. After inoculation, the fruits were placed in sterile petri dishes and placed on top of glass containers. The container was sealed and incubated at room temperature for 7 days. The decay incidence and disease index of the wolfberry fruits were measured using previous methods (Xu et al., 2016).

#### Effect of VOCs on wolfberry fruit postharvest natural decay

Untreated wolfberry fruits were treated with different numbers of LB plates, and after fumigation for 7 days at room temperature, the decay incidence and disease index were measured.

### Identification of VOCs

The method used for identifying VOC components of *B. velezensis* L1 is consistent with that described in our previous study (Ling et al., 2021). Then, 10 μl of the bacterial suspension of strain L1 was inoculated into a 20-mL headspace vial containing 5 ml of solid LB medium. The solid LB medium without the strain was used as a blank control, and each sample was repeated three times. After incubating the vials for 3 days in a 37°C incubator, the headspace-gas chromatography-ion mobility spectrometry (HS-GC-IMS) assay was performed. VOCs were collected and sent to the G.A.S. Department of Shandong HaiNeng Science Instrument Co., Ltd. (Shandong, China) for measurement using the HS-GC-IMS instrument (FlavourSpec^®^). The sample was incubated at 40°C for 15 min, and VOCs from the 500 μL headspace were injected into the testing instrument using a heated syringe (85°C). GC equipped with the MXT-5 column (15 m, ID: 0.53 mm df: 1 μm) was used for chromatographic separation at 60°C. Pure nitrogen (99.999% purity) was used as the carrier gas. The flow of drift gas (nitrogen) of the IMS was set to 150 ml/min, and a 9.8 cm drift tube was operated at a constant voltage at 45°C. The program was as follows: 2 ml/min for 2 min, 10 ml/min for 10 min, and 100 ml/min for 20 min. Data were acquired and processed using instrumental analysis software such as VOCal and Reporter, Gallery plot, Dynamic PCA, and GC × IMS Library. The GC-IMS Library Search software uses the National Institute of Standards and Technology database and IMS database for qualitative analysis of the components.

### *In vitro* responses of *Alternaria iridiaustralis* to a single component of VOC

The pathogenic fungus *A. iridiaustralis* was grown on PDA medium at 28°C for 7 days. Using a punching bear, a 6 mm-diameter disks were removed from the PDA plate’s edge and placed one by one in the center of each fresh petri plate. A sterile filter paper (diameter: 6 mm) was placed in the center of the petri dish lid. Then, equal volumes of each individual pure VOC (Table 1) were added to increase the air concentration from 20 μl/L to 100 μl/L. An identical volume of SDW was used for the control. A Vernier caliper was used to measure the plug diameter before the petri dishes were sealed and grown for 4 days at 28°C.

**Table 1:** Pure components comprising the VOCs of *Bacillus velezensis* L1, these substances were purchased for further experiments.

Compound	CAS	Source	Purity
2,3-butanedione	C431038	Macklin	≥99.0%
1-Hydroxy-2-propanone	C116096	Macklin	≥95.0%
Acetoin	C513860	Macklin	≥97.0%
2-pentanone	C107879	Macklin	≥99.0%
2-heptanone	C110430	Macklin	≥99.7%
Cyclohexanone	C108941	Macklin	≥99.0%
methyl 2-methylbutanoate	C868575	Macklin	≥98.0%
2-Pentylfuran	C3777693	Macklin	≥98.0%
2-methylpropyl butanoate	C539902	Macklin	≥98.0%

### *In vivo* control of *Alternaria iridiaustralis* on wolfberry fruit by pure synthetic components of VOCs

A single VOC component was chosen for additional testing *in vivo* based on the outcomes of *in vitro* testing. Fruits were infected and placed in a sterile petri plate. Then, the decay incidence and disease index of wolfberry fruit was determined with various quantities of pure components.

### Broad antagonistic activity of strain L1

We determined whether strain L1 has broad-spectrum antifungal activity against other significant fungal infections. Twelve pathogens were used in our test, namely *P. capsici*, *C. capsici*, *F. oxysporum*, *B. cinerea*, *R. solani*, *F. graminearum*, *F. annulatum*, *T. tumuli*, *C. fioriniae* and *F. arcuatisporum*. The following formula was used to compute the percentage of mycelial growth inhibition:

Inhibition rate (%) = [(dc − dt)/dc] × 100

Where the terms dc (cm) represent the average colony diameters of the control and treatment groups, respectively.

### Statistical analysis

SPSS 20.0 software was used to perform statistical analyses. Followed by the Duncan’s test, *p* < 0.05 was set to indicate a statistically significant difference. Drawn with the Origin 9.0 software, by measuring three independent replicates, all data were reported as the mean ± standard error.

## Results

### Identification of strain L1 using morphological and molecular markers

**Figure 1:**
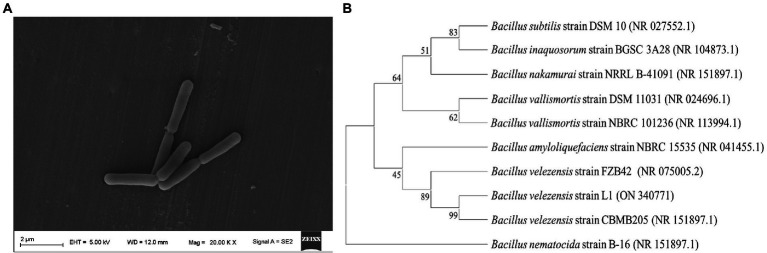
**(A)** Morphological characteristics of *B. velezensis* L1 detected by a scanning electron microscope. **(B)** Neighbor-joining tree of the *B. velezensis* L1 and its closely related species with the MEGA-X and 16S ribosomal RNA-sequencing data. The numbers at the nodes support the values derived from the MEGA-X bootstrap analysis.

An endophyte (strain L1) was obtained from *L. maackii* (Rupr.) Maxim (Figure 1A). The strain L1 exhibited phenotypic similarity to the *Bacillus* spp. with respect to the biochemical, morphological, Gram staining, and cultural characteristics. Nucleotide blast on NCBI revealed that strain L1 shared extremely high similarity with *Bacillus velezensis* species (99%). On the basis of the strain L1 16S rRNA’s sequence and blast alignment results, MEGA-X was employed to construct a phylogenetic neighbor connection tree that reflected the evolutionary relationship for L1 (Figure 1B). Combining all the resulting analyses, the isolate was identified to be *B. velezensis* (GenBank Accession No. ON340771).

### *In vitro* antifungal activity of VOCs

**Figure 2:**
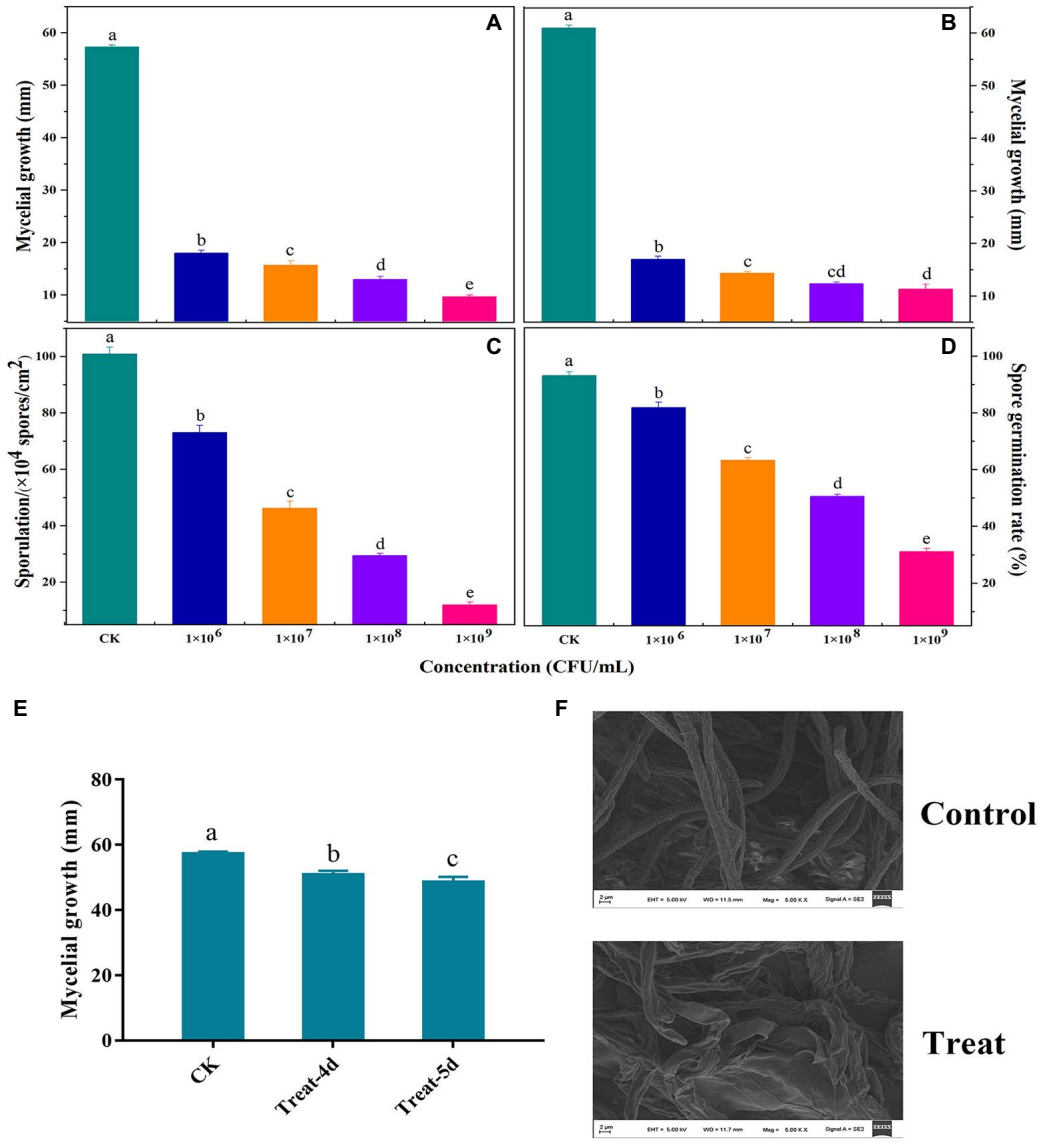
Antifungal activity of volatile organic compounds (VOCs) released by *B. velezensis* L1 against *A. iridiaustralis in vitro*. LB plates were coated with 80 μl of bacterial suspension at the concentration of 10^6^, 10^7^, 10^8^, 10^9^ colony forming units (CFU)/ml and then incubated at 37°C for 2 days. Their lids were subsequently replaced with PDA plates containing a 6 mm-diameter fungal disk or 20 μl of spore suspension of *A. iridiaustralis*. These plates were incubated at 28°C for 7 days. **(A)** Mycelial growth inhibition of a fungal disk of *A. iridiaustralis* on PDA plates exposed to VOCs at different concentrations. Different letters in represent differences (*p*  < 0.05) among groups. **(B)** Mycelial growth inhibition 20 μl of spore suspension of *A. iridiaustralis* on PDA plates exposed to VOCs. Effect of VOCs produced by *B. velezensis* L1 suspension at different concentrations on sporulation **(C)** and spore germination **(D)** of *A. iridiaustralis*. Data are presented as means ± SE with three replicates. **(E)** Mycelial of *A. iridiaustralis* was fumigated with VOCs for 4 and 5 days, then a 6-mm-diameter fungal disk was inoculated on fresh PDA plates, after incubated at 28°C for 7 days, the diameter of the mycelium was measured. **(F)** Scanning electronic micrographs of *A. iridiaustralis* in the absence and presence of the VOCs produced by *B. velezensis* L1 strain.

Figure 2 presents the findings related to the antifungal activity of VOCs produced by *B. velezensis* L1. Mycelial development, spore germination, and sporulation ability of A. iridiaustralis were significantly (*p* < 0.05) inhibited by VOCs produced by strain L1. An increased inoculum concentration of *B. velezensis* L1 improved VOC-induced inhibition. Furthermore, increasing the concentration also enhanced the inhibitory effect of VOCs on pathogenic fungi. The mycelial growth of plug and spore suspensions was inhibited by the bacterial suspension at 1 × 10^9^ CFU/ml by approximately 92.86 and 90.30%, respectively (Figures 2A,B); the spore germination and sporulation processes were inhibited by 33.11 and 12.04%, respectively (Figures 2C,D). *A. iridiaustralis* fumigated with VOCs grew more slowly than usual, as observed in (Figure 2E). The hyphal morphology of pathogenic fungi are dramatically altered by VOCs, as observed through SEM. The treatment group’s hyphae were twisted, flattened, enlarged, and lost their linearity compared with the control group (Figure 2F).

### *In vivo* biocontrol effects of VOCs

**Figure 3:**
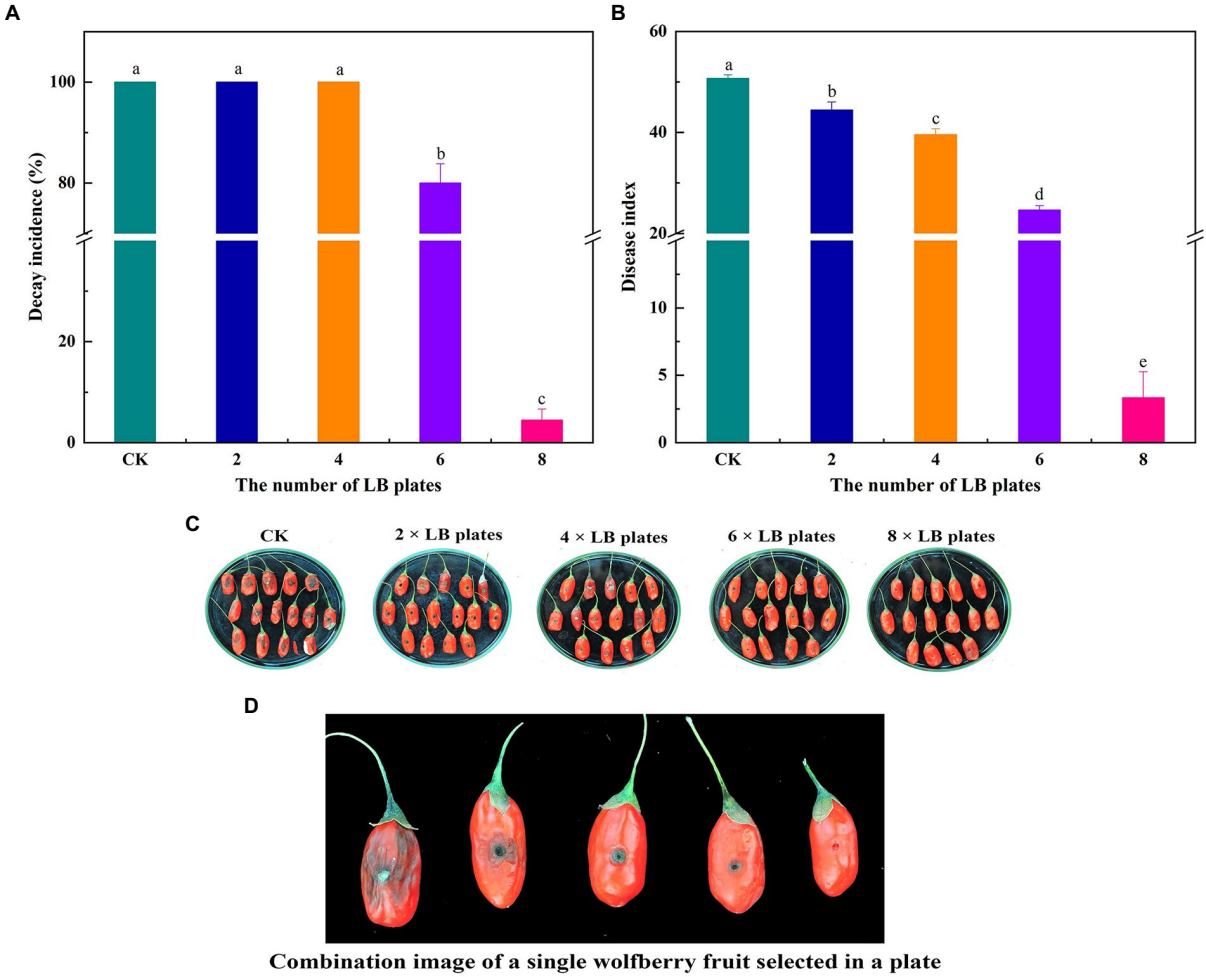
Fruits were fumigated with 2, 4, 6 and 8 LB plates, respectively, and the decay incidence **(A)** and disease index **(B)** were calculated according to the disease severity of the fruit infected with *A. iridiaustralis*. Biocontrol of wolfberry rot by biofumigation with different numbers of LB plates **(C)**. Combination image of a single wolfberry fruit selected in a plate **(D)**. Columns with different lower-case letters within the same panel are significantly different at *p* < 0.05 level according to Duncan’s multiple range test.

To investigate the impact of VOC concentration on the effectiveness of biocontrol ability of VOC *in vivo*, various numbers of LB plates containing viable cells were used. VOCs considerably impeded the growth and spread of *A. iridiaustralis* (Figure 3). No mycelial was observed growth on the wolfberry fruits after exposure to the 8 LB plates. The decay incidence and disease index of the treatment group were 4.45 and 3.33%, respectively, which were considerably lower than those of the control group (100 and 50.67%, respectively). In general, biological control effects were better at higher VOC concentrations.

### Effect of VOCs on postharvest natural decay

**Figure 4:**
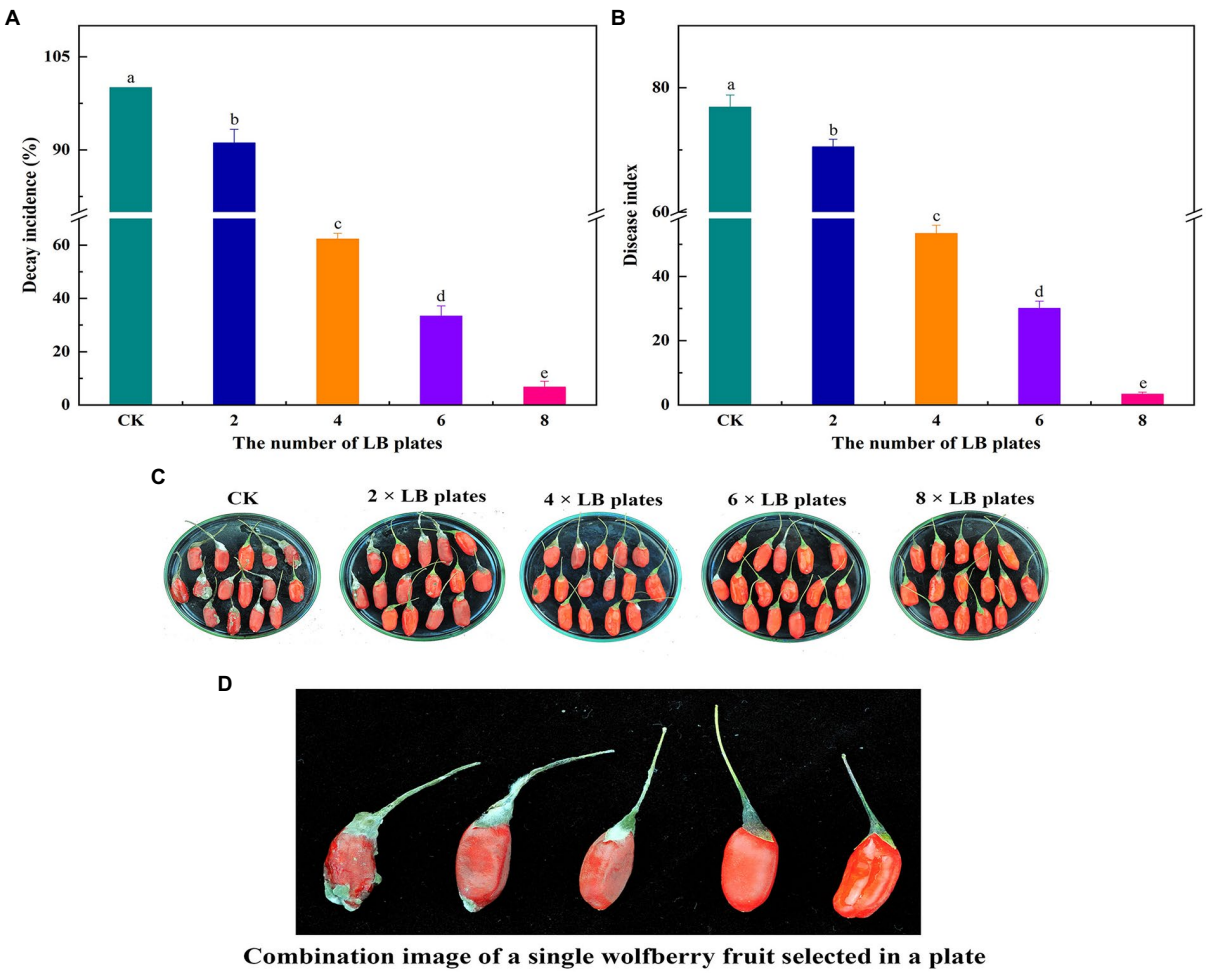
2, 4, 6 and 8 LB plates were used to fumigate untreated wolfberry fruit. The container were sealed and followed by incubation at room temperature for 7 days, the decay incidence **(A)** and disease index **(B)** were measured. Biocontrol of VOCs produced by *B. velezensis* L1 on postharvest natural decay of wolfberry fruit **(C)** and combination image of a single wolfberry fruit selected in a plate **(D)**. Columns with different lower-case letters within the same panel are significantly different at *p* < 0.05 level according to Duncan’s multiple range test.

Figure 4 demonstrates the outcomes of the impact of VOCs on the fruit’s natural decay after harvest. Under natural circumstances, the VOCs generated by *B. velezensis* L1 had a larger inhibitory effect on the decay incidence of postharvest wolfberry as the number of LB plates increased. The best inhibitory effect was observed when 8 LB plates were used, the wolfberry fruit was intact, and the lowest disease index was 3.33.

### HS-GC-IMS identification of VOCs

**Figure 5:**
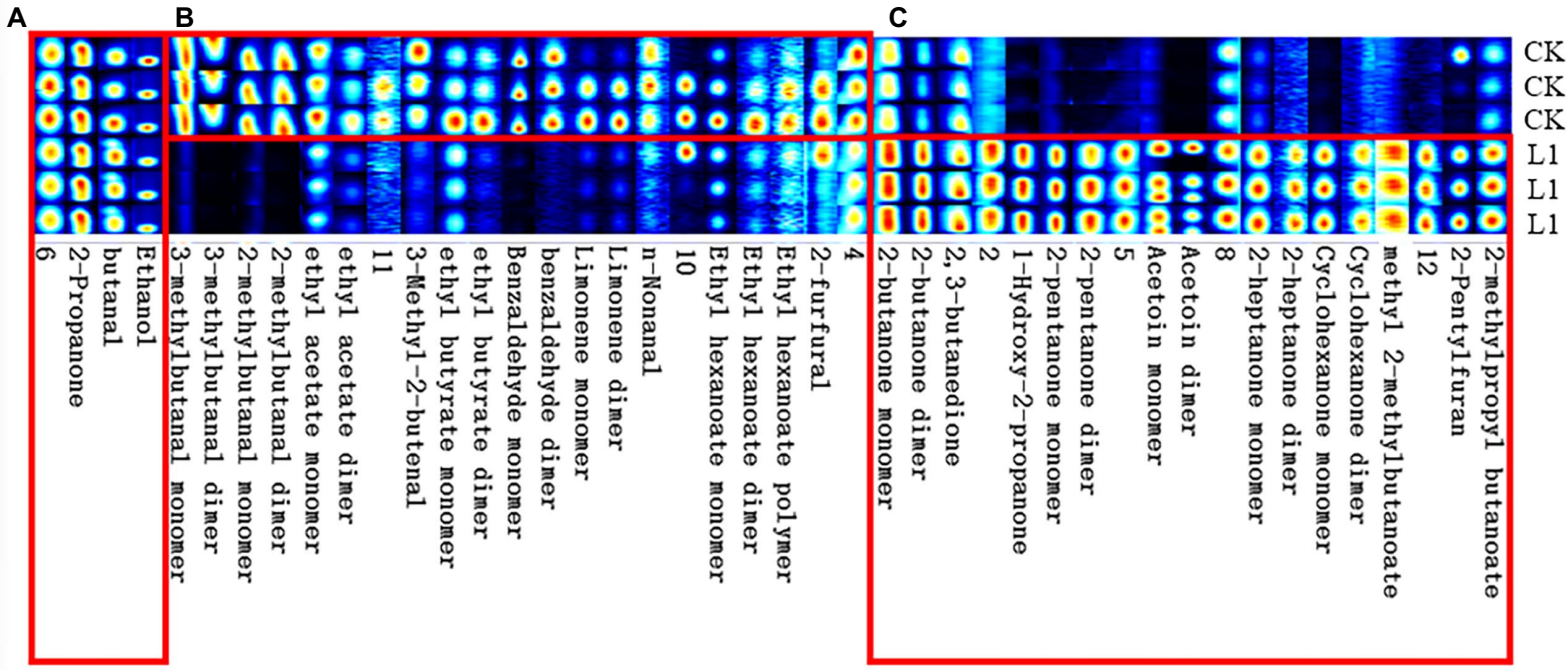
The Gallery plot of selected VOCs in the gas-phase ion mobility spectrum. Each row in the figure represents that all the signal peaks were selected in one sample, and each column represented the signal peaks of the same VOCs in different samples. Substances in area **(A)** were both identified in CK and *B. velezensis* L1. Substances in area **(B)** were identified in CK. Substances in area **(C)** were identified in *B. velezensis* L1.

From Figure 5, the whole VOC information of each sample as well as the variations in VOCs across samples can be observed when each row represents all signal peaks selected in a single sample and each column represents the signal peaks of the same VOC in numerous samples. The chemical’s concentration is qualitatively represented by the hues of the peaks; the higher the red color, the higher the concentration. Table 2 lists a collection of specific details. Strain L1 produced the following recognized VOCs: 2-butanone, 2,3-butanedione, 1-hydroxy-2-propanone, 2-pentanone, acetoin, 2-heptanone, cyclohexanone, methyl 2-methylbutanoate, 2-pentylfuran, and 2-methylpropyl butanoate.

**Table 2:** Qualitative information from HS-GC-IMS analysis of VOCs from tested *B. velezensis* L1.

**Count**	**Compound**	**CAS#**	**Formula**	**MW**	**RI**	**Rt [sec]**	**Dt [RIPrel]**
1	2-butanone monomer	C78933	C4H8O	72.1	608.5	139.894	1.0597
2	2-butanone dimer	C78933	C4H8O	72.1	601.6	136.916	1.24743
3	2,3-butanedione	C431038	C4H6O2	86.1	599	135.826	1.17873
4	1-Hydroxy-2-propanone	C116096	C3H6O2	74.1	638.4	152.575	1.22642
5	2-pentanone monomer	C107879	C5H10O	86.1	696	180.037	1.12034
6	2-pentanone dimer	C107879	C5H10O	86.1	697.7	181.4	1.37338
7	Acetoin monomer	C513860	C4H8O2	88.1	723.1	202.308	1.05484
8	Acetoin dimer	C513860	C4H8O2	88.1	720.3	200.04	1.32941
9	2-heptanone monomer	C110430	C7H14O	114.2	896.6	378.474	1.26411
10	2-heptanone dimer	C110430	C7H14O	114.2	895.7	376.962	1.62921
11	Cyclohexanone monomer	C108941	C6H10O	98.1	903	389.726	1.15422
12	Cyclohexanone dimer	C108941	C6H10O	98.1	902.6	389.001	1.45337
13	methyl 2-methylbutanoate	C868575	C6H12O2	116.2	775.6	245.523	1.18758
14	2-Pentylfuran	C3777693	C9H14O	138.2	1002.5	564.479	1.25597
15	2-methylpropyl butanoate	C539902	C8H16O2	144.2	960.2	489.067	1.32923

Dt, drift time; MW, molecular mass; RI, retention index; Rt, retention time; VOC, volatile organic compound.

### *Alternaria iridiaustralis* is inhibited by a single known VOC

The effects of nine components on the growth of *A. iridiaustralis* mycelium were determined (Table 3). Among the nine tested pure substances, only 2,3-butanedione (40 μl/L) showed a strong effect, completely inhibiting the growth of the *A. iridiaustralis* mycelium. The remaining substances, including 1-hydroxy-2-propanone, 2-pentanone, acetoin, 2-heptanone, cyclohexanone, methyl 2-methylbutanoate, 2-pentylfuran and 2-methylpropyl butanoate, showed very weak or no antifungal activity on PDA plates.

**Table 3:** Effects different concentrations of tested compounds on the mycelial growth of *A. iridiaustralis* after an incubation at 28°C for 4 d.

Volatile compound	Different concentrations of volatiles				
	20 μl/L	40 μl/L	60 μl/L	80 μl/L	100 μl/L
Control	35.7 ± 0.4^a^	35.7 ± 0.4^a^	35.7 ± 0.4^a^	35.7 ± 0.4^a^	35.7 ± 0.4^a^
2,3-butanedione	29.9 ± 0.5^c^	19.1 ± 0.4^b^	06.0 ± 0.0^c^	06.0 ± 0.0^c^	06.0 ± 0.0^d^
1-Hydroxy-2-propanone	34.5 ± 0.4^a^	34.1 ± 0.4^a^	33.9 ± 0.4^b^	33.6 ± 0.5^b^	33.0 ± 0.3^c^
Acetoin	35.5 ± 0.4^a^	35.4 ± 0.4^a^	35.9 ± 0.3^a^	35.1 ± 0.2^ab^	34.7 ± 0.4^ab^
2-pentanone	35.5 ± 0.3^a^	34.3 ± 0.4^a^	34.5 ± 0.4^ab^	34.2 ± 0.2^b^	33.9 ± 0.3^bc^
2-heptanone	31.4 ± 0.4^b^	31.7 ± 0.3^b^	32.9 ± 0.2^b^	33.7 ± 0.3^b^	32.6 ± 0.4^c^
Cyclohexanone	35.9 ± 0.4^a^	35.8 ± 0.5^a^	35.6 ± 0.2^a^	35.9 ± 0.3^a^	36.1 ± 0.5^a^
methyl 2-methylbutanoate	35.9 ± 0.4^a^	34.1 ± 0.4^a^	33.2 ± 0.2^b^	34.5 ± 0.4^ab^	32.6 ± 0.5^c^
2-Pentylfuran	34.1 ± 0.2^a^	35.3 ± 0.4^a^	34.4 ± 0.4^ab^	34.1 ± 0.4^b^	34.1 ± 0.2^bc^
2-methylpropyl butanoate	34.8 ± 0.2^a^	34.1 ± 0.5^a^	34.4 ± 0.4^ab^	33.9 ± 0.3^b^	34.2 ± 0.3^bc^

Each value is the mean of three replicates ± standard error. Values in a column followed by a different letter are significantly different according to Duncan’s multiple range test at *p* < 0.05 level.

### Effects of pure VOC chemicals against *Alternaria iridiaustralis* on wolfberry

As shown in Table 4, 2,3-butanedione was found to be an effective BCA. At 60 μl/L, it completely inhibited the growth of *A. iridiaustralis*, and no obvious lesions were found on the wolfberry fruits. The decay incidence and disease index decreased significantly.

**Table 4:** Effects different concentrations of 2,3-butanedione on the wolfberry fruit decay.

Concentrations (μL/L)	Decay incidence (%)	Disease index
0	100.00 ± 0.00^a^	49.63 ± 1.96^a^
20	92.59 ± 3.70^b^	22.50 ± 1.44^b^
40	48.15 ± 3.71^c^	7.83 ± 1.17^c^
60	0.00 ± 0.00^d^	0.00 ± 0.00^d^
80	0.00 ± 0.00^d^	0.00 ± 0.00^d^
100	0.00 ± 0.00^d^	0.00 ± 0.00^d^

Each value is the mean of three replicates ± standard error. Values in a column followed by a different letter are significantly different according to Duncan’s multiple range test at *p* < 0.05 level.

### Broad spectrum antifungal activity of volatiles from *Bacillus velezensis* L1

**Figure 6:**
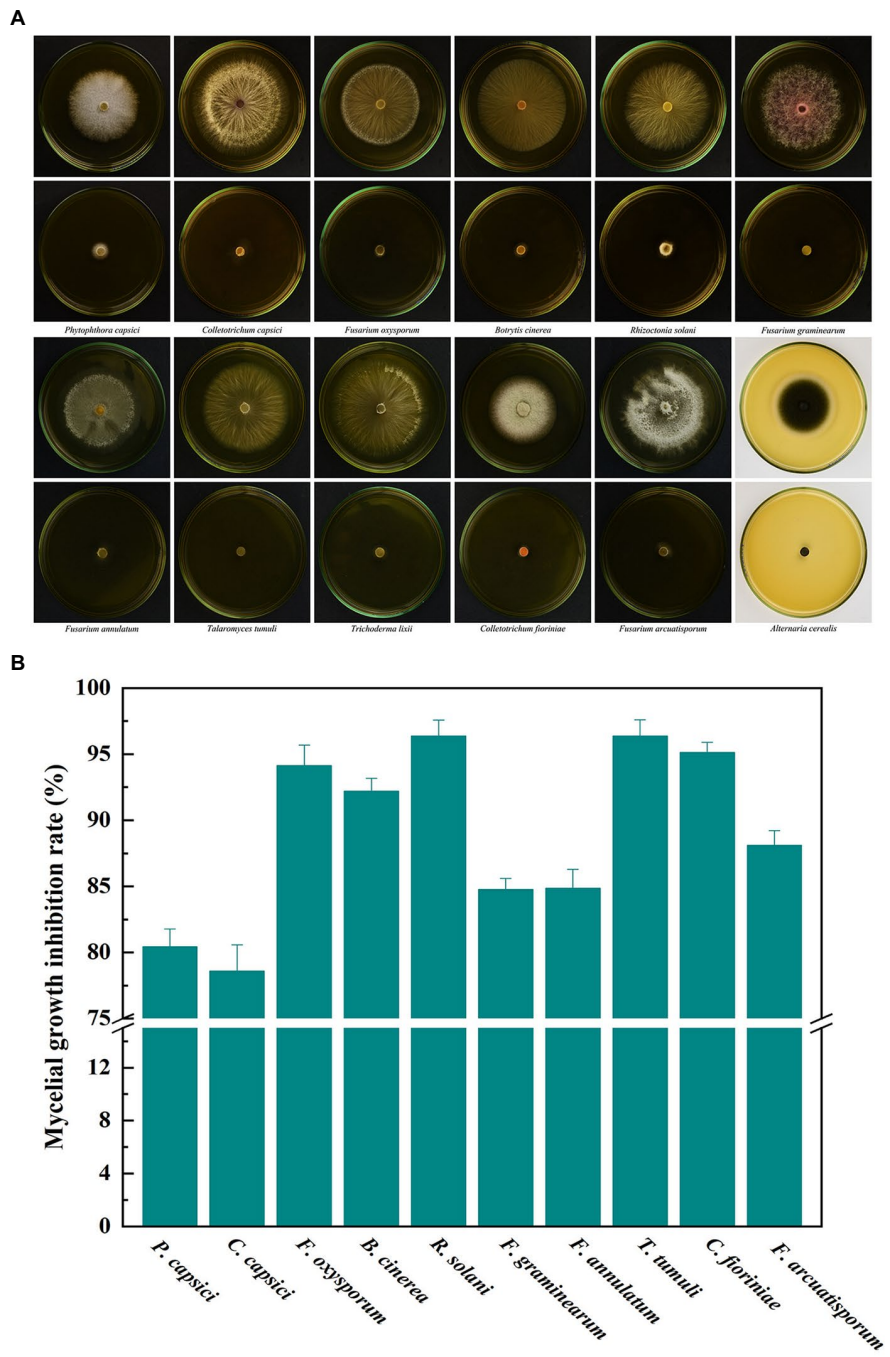
**(A)** Broad antifungal activity of *B. velezensis* L1 against ten fungal pathogens. **(B)** Corresponding histograms for statistical data on mycelial growth inhibition rate. Different fungal strains including *P. capsici*, *C. capsici*, *F. oxysporum*, *B. cinerea*, *R. solani*, *F. graminearum*, *F. annulatum*, *T. tumuli*, *C. fioriniae* and *F. arcuatisporum* were used in the tests.

Ten important pathogenic fungi including *P. capsici*, *C. capsici*, *F. oxysporum*, *B. cinerea*, *R. solani*, *F. graminearum*, *F. annulatum*, *T. tumuli*, *C. fioriniae* and *F. arcuatisporum*, were used to test the broad antifungal activity of *B. velezensis* L1. In the strain L1 treatment, the mycelial growth of the pathogenic fungi was greatly inhibited (Figure 6). The inhibition rate of L1 strain against the nine pathogenic strains was >80%. These results suggest that active volatiles produced by *B. velezensis* L1 have a broad antifungal activity against different genera of pathogenic fungi.

## Discussion

Postharvest regulation of fruits and vegetables can result in an incredibly high value (Lemos Junior et al., 2020). Recent VOCs control research has produced positive results and is regarded as a useful alternative strategy for reducing fungal infection (Raza et al., 2016; Syed-Ab-Rahman et al., 2019). In this study, we isolated and characterized a strain of *B. velezensis* L1 that suppresses *A. iridiaustralis* growth in wolfberry during the postharvest process and displayed the broad-spectrum antifungal activity of VOCs obtained from *B. velezensis* L1. Therefore, *B. velezensis* L1 is anticipated to be employed as an antagonistic microbe. To the best of our knowledge, this is the first known study exhibiting the antagonistic activity of *B. velezensis* VOCs against *A. iridiaustralis* on postharvest wolfberry.

*In vitro* antifungal experiments revealed that VOCs produced by *B. velezensis* L1 strongly inhibited the growth of *A. iridiaustralis* mycelium, spore germination, and sporulation. VOCs generated by *B. velezensis* C16 significantly suppress *A. solani* mycelium development and spore germination (Zhang et al., 2021). According to Jiang et al. (2018), VOCs from the *B. velezensis* strain may prevent sporulation of *B. cinerea* in peppers. This agrees with the findings obtained for *B. velezensis* L1. Additionally, the regrowth of *A. iridiaustralis* was reduced by the VOCs from *B. velezensis* L1. The effects of VOCs on *B. cinerea* were comparable to those of *Pseudomonas fluorescens* ZX (Zhong et al., 2021). This might be because VOCs disrupt the pathogenic fungi’s natural morphological structure and interfere with their normal proliferation. However, the damage appears to be lost with a reduction in VOC concentration, possibly because of the robust recovery and reproductive capacity of the fungi. According to SEM analysis, the VOCs from *B. velezensis* L1 can severely damage the morphology of *A. iridiaustralis*. Numerous studies have demonstrated that VOCs can harm pathogenic the walls and membrane systems of pathogenic fungi, impairing their ability to perform essential tasks (Zhao et al., 2019; Wang et al., 2021b). This might be one of the key ways through which VOCs prevent harmful the growth of fungi.

The effectiveness of VOCs *in vivo* was further investigated in light of the superior inhibitory capacity of *B. velezensis* L1 *in vitro*. The findings demonstrated that VOCs from *B. velezensis* L1 can greatly reduce disease severity in wolfberry. Although many descriptive studies have investigated the effectiveness of VOCs (Archana et al., 2021; Wang et al., 2021a), more research is focused on the screening of strains and the identification of volatiles produced, with most authors not choosing to explore the relationship between their concentrations and diseases, which may be due to difficulties associated with testing. Calvo et al. (2020) reported that the *B. velezensis* strain inhibits mycelial growth and sporulation and is not pathogenic to humans after use. However, the VOCs concentration was not considered in their study. Myo et al. (2019) found that *B. velezensis* NKG-2 adversely affected the growth of six pathogenic fungi, but they too did not consider the VOCs concentration. The effect of VOC concentrations on the prevention, occurrence, and severity of diseases is of great value for the postharvest preservation of fruits and vegetables (Sharifi and Ryu, 2016). These concentrations were influenced by different numbers of LB plates. With an increase in concentration, the disease status of wolfberry fruits inoculated with the pathogenic fungus *A. iridiaustralis* changed significantly. Wang et al. (2021c) also confirmed that a certain concentration of VOCs produced by *P. fluorescens* ZX can significantly control gray mold in citrus and inhibit decay development. When VOCs reach a certain concentration, the growth of pathogenic fungi in fruits is completely inhibited, resulting in the best control effect. This suggests that it is entirely feasible to utilize VOCs produced by antagonistic microorganisms as potential BCAs. More importantly, VOCs also have an excellent control effect on fruits that rot naturally after harvest, inhibited all pathogenic fungi that caused the disease, which greatly prolongs the storage time of wolfberry. Studies have confirmed that VOCs produced by some bacteria, yeast and fungi can induce resistance against pathogens in fruits and vegetables (Zheng et al., 2019). This may be related to another antifungal mechanism of VOCs. Zhou et al. (2019) reported that the VOCs of strain *CF*-3 could reduce the enzymatic activity involved in fruit decomposition, activate the antioxidant enzymes to prevent cell damage, and elevate the disease-resistant enzyme activity to prevent the invasion of pathogenic fungi, thereby inducing resistance to fungi. Lee et al. (2019) showed that VOCs released by microorganisms induce self-resistance in the host to avoid the pathogen-induced harm. For the control of postharvest natural decay, strain L1 can be used as a new preservative with significant effect, which is of great value in commercial production.

In general, antagonistic microorganisms can produce VOCs such as ketones, alcohols, aldehydes, esters, and acidic and aromatic compounds with antifungal activity, and different strains released VOCs are specific (Raza et al., 2015; Kai, 2020). In addition, VOCs can be altered by changes in culture conditions. For example, *P. fluorescens* ZX produced different VOCs when incubated on NB or NA (Wang et al., 2020). *B. amyloliquefaciens* PP19, *Exiguobacterium acetylicum* SI17, *B. pumilus* PI26 were reported to vary greatly in their VOCs with incubation time and between strains (L. Zheng et al., 2019). The FlavourSpec^®^ Flavor Analyzer, which uses HS-GC-IMS technology, is mainly used for the research of food flavor (Wang et al., 2019; Yuan et al., 2019). Because of the high sensitivity and high resolution, the HS-GC-IMS technique is effective in identifying VOCs released by different strains. In this study, based on the complexity and diversity of VOCs, volatile gas chemicals emitted by *B. velezensis* L1 were identified using HS-GC-IMS, and 10 main components were found. The components identified varied from the volatile components discovered in other studies, which could be due to variations in the growth environment of the hostile bacteria (Wallace et al., 2017). On the other hand, the results obtained may vary because of the use of different types of equipment and materials in experiments to identify VOC components.

Nine pure compounds were commercially available and selected for further analysis. Among the components identified, 2,3-butanedione had the strongest inhibitory effect on pathogenic fungi both *in vitro* and *in vivo*. 2,3-Butanedione could reduce the rot disease index of wolfberry fruit. Compared with the complex VOCs components, pure compounds have clear structural characteristics and are easy to produce and synthesize and may have practical applications in future. Calvo et al. (2020) showed that 20 μl/L of 2,3-butanedione could completely control gray mold in grapes and reduce blue rot in mandarins to 60%. Gergolet Diaz et al. (2021) showed that 2,3-butanedione had the strongest inhibitory effect on the mycelial growth of *F. verticillioides*. 2,3-Butanedione is a naturally occurring, safe and edible volatile alpha-diketone often used in food additives and flavors because of its good flavor. Pathogenic fungi are very sensitive to even very low concentrations of VOC, and therefore, this VOC is considered a promising compound for postharvest preservation (Zheng et al., 2020). 2-Pentanone, 2-heptanone, and acetoin are the most common and abundant VOCs in many strains (Wu et al., 2015; Lazazzara et al., 2021). As observed in our study, they have little impact on the hyphal development of pathogenic fungi. However, VOCs are usually complex in composition, and their inhibitory effects often do not depend on a single active ingredient (Wang et al., 2021c). Few studies have investigated the optimal application and activity of the combination of VOC. Thus, whether the main components in these VOCs are synergistically involved in the antifungal effect deserves further investigation. 1-Hydroxy-2-propanone, cyclohexanone, methyl 2-methylbutanoate, 2-pentylfuran and 2-methylpropyl butan were found to be produced by the *B. velezensis* strain for the first time. However, they showed no antifungal activity against *A. iridiaustralis* in the tests. This could be related to the concentration chosen, some antifungal compounds showed weak activity or were ineffective at low concentrations (Vicentini et al., 2007). However, it is worth considering that high concentrations of antifungal substances can harm the human body, which is not in line with our original intention.

In addition, tests on pathogenic fungi that cause severe diseases in crops, fruits, vegetables and other foods have found that VOCs from *B. velezensis* L1 also significantly inhibit its growth. This provides more possibilities for the application of *B. velezensis* L1 and its VOCs as preservatives for controlling these diseases during storage. For different pathogenic fungi, the inhibitory effect of VOCs is also different. At present, the antifungal mechanisms discovered by VOCs mainly include inhibition of mycelial growth, damage to cell walls, regulation of enzyme activities, and induction of host resistance (Zhong et al., 2021). It is still important to explore the interactions and connections between VOCs, pathogenic fungi and hosts.

This work is the first to investigate the inhibitory effect of *B. velezensis* L1 on postharvest disease of wolfberry. In experiments, *B. velezensis* L1 and its volatiles (2,3-butanedione) can effectively reduce the decay caused by pathogenic fungi and prolong storage time. These results provide favorable evidence for the biocontrol activity of strain L1 against *A. iridiaustralis* and other important fungal pathogens, and also provides insights to develop storage systems for fresh fruits and vegetables after harvest.

## Data availability statement

The original contributions presented in the study are included in the article/supplementary material, further inquiries can be directed to the corresponding author.

## Author contributions

All authors contributed to the study conception and design. Material preparation, data collection, and analysis were performed by HL, CY, and LL. The first draft of the manuscript was written by HL. All authors contributed to the article and approved the submitted version.

## Funding

The Lanzhou Science and Technology Plan Project 2018-1-104 is sincerely acknowledged by the authors; Special Fund Project for Guiding Science and Technology Innovation and Development in Gansu Province 2019ZX-05. Higher Education Industry Support Plan of Gansu Province (2020C-21).

## Conflict of interest

The authors declare that the research was conducted in the absence of any commercial or financial relationships that could be construed as a potential conflict of interest.

## Publisher’s note

All claims expressed in this article are solely those of the authors and do not necessarily represent those of their affiliated organizations, or those of the publisher, the editors and the reviewers. Any product that may be evaluated in this article, or claim that may be made by its manufacturer, is not guaranteed or endorsed by the publisher.
